# Geographical Discrimination of Bell Pepper (*Capsicum annuum*) Spices by (HS)-SPME/GC-MS Aroma Profiling and Chemometrics

**DOI:** 10.3390/molecules26206177

**Published:** 2021-10-13

**Authors:** Samantha Reale, Alessandra Biancolillo, Chiara Gasparrini, Luciano Di Martino, Valter Di Cecco, Aurelio Manzi, Marco Di Santo, Angelo Antonio D’Archivio

**Affiliations:** 1 Dipartimento di Scienze Fisiche e Chimiche, Università degli Studi dell’Aquila, Via Vetoio, 67100 L’Aquila, Italy; samantha.reale@univaq.it (S.R.); alessandra.biancolillo@univaq.it (A.B.); chiara.gasparrini@student.univaq.it (C.G.); 2 Majella Seed Bank-Parco Nazionale della Majella, Via Badia 28, 67039 Sulmona, Italy; luciano.dimartino@parcomajella.it (L.D.M.); valter.dicecco@univaq.it (V.D.C.); marco.disanto@parcomajella.it (M.D.S.); 3 Independent Researcher, 66010 Gessopalena, Italy; aureliomanzi1963@gmail.com

**Keywords:** bell pepper spices, *Capsicum annum*, HS-SPME, GC-MS, classification, PLS-DA, geographical origin, variable selection, VIP

## Abstract

Dried and ground red pepper is a spice used as seasoning in various traditional dishes all over the world; nevertheless, the pedoclimatic conditions of the diverse cultivation areas provide different chemical characteristics, and, consequently, diverse organoleptic properties to this product. In the present study, the volatile profiles of 96 samples of two different ground bell peppers harvested in diverse Italian geographical areas, Altino (Abruzzo) and Senise (Lucania), and a commercial sweet paprika, have been studied by means of headspace solid-phase microextraction (HS-SPME) coupled with gas chromatography-mass spectrometry (GC-MS). The investigation of their volatile profile has led to the identification of 59 analytes. Eventually, a discriminant classifier, Partial Least Squares Discriminant Analysis (PLS-DA), was exploited to discriminate samples according to their geographical origin. The model provided very accurate results in external validation; in fact, it correctly classified all the 30 test samples, achieving 100% correct classification (on the validation set). Furthermore, in order to understand which volatiles contribute the most at differentiating the bell peppers from the different origins, a variable selection approach, Variable Importance in Projection (VIP), was used. This strategy led to the selection of sixteen diverse compounds which characterize the different bell pepper spices.

## 1. Introduction

Bell peppers are the fruits of *Capsicum annum* (Solanaceae) species, widely cultivated in Africa, Asia, and Mediterranean countries, used worldwide as vegetables, condiments, and spices. Capsicum peppers are a good source of phytochemicals, including capsacinoids, phenolic compounds, carotenoids, vitamins C and E, which provide important health benefits [1,2]. Dehydration processing of bell pepper fruits is practiced all over the world to increase the storage time and convert this vegetable into value-added products, utilized as key ingredients and spices in traditional gastronomy or as natural coloring/flavoring agents in various preparations in the food industry. The term paprika, the word for bell pepper in some East European languages, is also commonly adopted to identify the Capsicum spice. Composition and organoleptic properties (color, taste, and aroma) of bell pepper spice are dependent on the *Capsicum annum* L. cultivar, the pedoclimatic conditions of the cultivation site, the cultivation practices, and the kind of dehydration process (sun-drying, oven-drying, or smoking) adopted locally [3,4]. 

It has been documented that spices and herbs are particularly susceptible to adulteration or origin’s mislabeling since their supply chain tends to be long, complex, and can pass through many countries, providing several occasions for criminals to commit fraudulent practices [5]. Unlike detection of adulterants (illegal artificial colorants or bulking agents), false origin labelling or contamination of origin-certified spices with low-quality products cultivated elsewhere cannot be unveiled by conventional targeted analytical methods due to the lack of specific markers directly related to the product origin [6]. Various fingerprinting or profiling methods, based on vibrational spectroscopies [7,8], high- or ultrahigh-performance liquid-chromatography coupled to different detector systems [9,10,11,12], and energy dispersive X-ray fluorescence [13], have been proposed to identify the origin of bell pepper spices. In this context, it has been found that the profiles of phenolic acids, polyphenolic compounds and capsacinoids are promising indicators for geographical traceability purposes.

The Italian pepper germplasm consists of highly diverse landraces selected locally across many years on the basis of a recognizable morphology and adapted to the local pedoclimatic conditions and the traditional cultivation practices [14]. In Peninsular Italy, the air- or sun-dried pepper fruits, often called “cruschi” (crispy) bell peppers, are commonly used to prepare traditional dishes and their successive oven-drying and grinding produces a spice that is also largely employed in traditional gastronomy and as a seasoning ingredient in typical cured meats. 

In the present work, we characterize Senise (Lucania, South Italy) and Altino (Abruzzo, Central Italy) bell pepper spices through the analysis of the aroma composition using gas-chromatography with mass spectrometry detection (GC/MS) preceded by headspace solid-phase micro-extraction (HS-SPME). The Senise sweet pepper variety, a locally widespread ecotype cultivated in various municipalities around the homonymous town, has been recognized as a Protected Geographical Indication (PGI) specialty since 1996. Dehydration of Senise bell peppers is carried out through the exposure of the fruits on strings or in the form of individual whole pods either to indirect sunlight or in well-ventilated drying rooms. Once the drying stage is over, the peppers to be ground can be heated in an oven to remove the residual moisture. The Altino bell pepper variety, locally called “a cocce capammonte” (with the head up) because the fruits grow pointing upwards, is cultivated in the Aventino and Sangro Valleys (Province of Chieti) and has been included by Slow Food Foundation in both Slow Food Presidia and Ark of Taste projects aimed at the safeguard of agricultural biodiversity and small–scale, family-based food production systems. The first document reporting the cultivation of Altino bell pepper is a notarial deed of 1752 [15]. The spice is obtained by grinding the air-dried bell peppers after these have been toasted in an oven.

To assess whether the *Capsicum annum* variety and the local cultivation and dehydration conditions confer peculiar sensorial properties to the flavor of Senise and Altino bell peppers, their aroma composition was also compared to that of a commercial sweet paprika with no specific indications concerning the geographical origin and manufacturing conditions. 

In food analysis, HS-SPME sampling has been often preferred to conventional extraction methods because it is less laborious, requires a smaller amount of sample, and can avoid the decomposition of thermolabile constituents [16,17,18]. As for bell pepper spice, HS-SPME/GC-MS was previously applied to assess the relationship between the aroma and the dehydration conditions [3,4] and to investigate the evolution in the volatile composition of the spice upon heating [19]. HS-SPME/GC-MS was also used to identify the volatile constituents of ripe fruits of *Capsicum annum* accessions with different geographical origins [20,21]. In addition, to discriminate different Italian cultivars, HS-SPME/GC-MS aroma profiling was performed on the bell pepper samples after oven-drying and grinding carried out in the laboratory under controlled conditions [22]. To the best of our knowledge, however, HS-SPME/GC-MS has never been applied before to trace the origin of bell pepper spices with recognized geographical identity, whose aroma is expected to depend not only on the *Capsicum annum* cultivar but also on the dehydration procedure adopted locally in the final step of spice production. In the present work, an experimental design was preliminarily applied to optimize the HS-SPME conditions. Successively, the volatile profile of Altino and Senise bell pepper spices along with a commercial sweet paprika were collected under the optimized conditions, and a discriminant classifier named Partial Least Squares Discriminant Analysis (PLS-DA) [23] was used to evaluate the potentiality of the aroma composition to trace the geographical origin of this spice. The choice fell on this method because it allows an unambiguous classification of samples, it presents a number of advantages from the interpretation standpoint, and, in the literature, it has widely shown its suitability for the analysis of chromatographic data in the field of food chemistry [24,25,26,27,28,29,30].

## 2. Results and Discussion

### 2.1. Optimisation of the HS-SPME/GC-MS Analysis 

To optimize the experimental conditions related to the HS-SPME/GC-MS analysis, and, in particular, in relation to the exposure time (*t*) of the fiber and the temperature (*T*) of the sample, a design of the experiment 2^*k*^ was used. The tested levels were: 10 and 30 min for *t* and 90 and 70 °C for *T*. An additional experiment was carried out in triplicate in the central point (*T* = 80 °C, *t* = 20 min) to estimate the standard deviation associated to the measured response and evaluate the predictive ability of the response surface. A resume of the experiments and the design matrix are reported in Table 1.

Once the planned experiments had been carried out, the chromatographic peaks between 5 and 19 min were integrated, the ten peaks with the largest area were eliminated, and, eventually, the total area of the remaining peaks was calculated (*A*). The logarithm of this entity has been used as response for the creation of the regression model finalized to the optimization of the HS-SPME experimental condition aimed at the maximization of the global intensity of the minor volatile components.

The regression of the observed responses in the four points of the 2^2^ experimental design (reported in Table 1) against the investigated factors provided the following model.

(1) logA=5.749−0.0475t+0.132T−0.100tT

It can be noted that the predicted response in the central point of the experimental domain, which is represented by the intercept of the regression model (5.749), is very close to the measured response in this point (5.732 ± 0.035) supporting the excellent generalization ability of the model. By contrast, only a moderate agreement was observed when the unscaled total chromatographic area (*A*) was modelled. 

**Figure 1:**
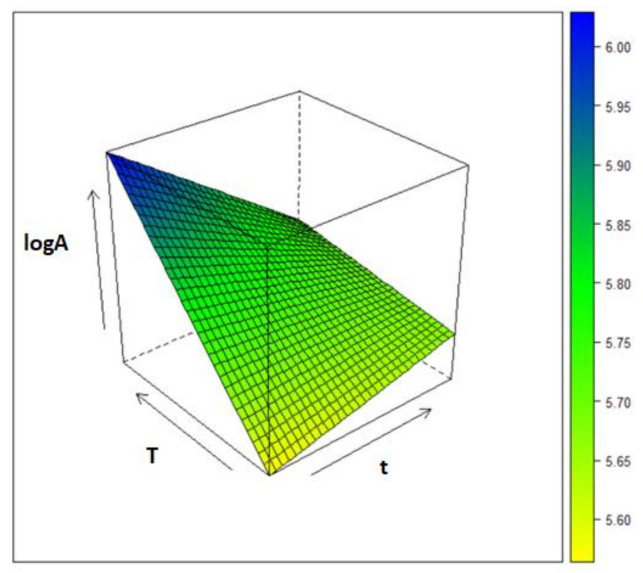
Response Surface associated to the experimental range investigated by the DoE.

Eventually, a response surface plot (Figure 1) was realized in order to define the optimal experimental conditions. As can be appreciated by Figure 1, log*A* is maximized when *T* is at the highest level, 90 °C, and the exposure time is at the lowest, corresponding to 10 min. In the light of these considerations, these conditions have been used for all the subsequent analyses. 

### 2.2. Volatile Profile by of Bell Pepper Spices by HS-SPME/GC-MS 

The bell pepper spices coming from Altino (Altino 1, 2, and 3), Senise, and a commercial paprika have been analyzed by HS-SPME/GC-MS under the optimized experimental conditions. The 59 volatile compounds identified in the GC/MS chromatograms along with the retention time (RT) and the mean relative (%) area in the analyzed samples are reported in Table 2. 

*β*-Ionone (peak n°43) is one of the most abundant compounds detected in the bell pepper spice aroma and it reaches the maximum abundance in Altino 1 sample (ca. 19%), while in paprika represents only about 6% of the total area. This compound derives from the degradation of *β*-carotene and gives intense floral and fruity notes [31]. As a whole, ten volatile compounds deriving from degradation of carotenoids [32] have been identified in the bell pepper samples (Table 2, compounds highlighted by “a” apex). The cumulative area provided by these compounds in the chromatograms reaches 59% in the case of Altino 1 spice, while the lowest relative content is observed in the Altino 2 and paprika samples (about 34% of the total area). Methyl benzaldehydes (namely 4-methyl benzaldehyde and 2,4-dimethylbenzaldehyde, peaks n°13 and 19, respectively) can be also tentatively considered as carotenoid degradation compounds on the basis of what was proposed by Rios et al. [21]. In addition, two dienones, namely 3,5-octadien-2-one and 3,5-nonadien-2-one (peak n°12 and 14), which have the same skeleton of 6-methyl-3,5 dien-2-ones except for the methyl branching in carbon 6, may be also originated by carotenoid degradation. 

Another interesting class of odor active components of the bell pepper spice aroma originates from non-enzymatic thermal degradation reactions such as the Maillard reaction and Strecker degradation, whose main products, the so-called Strecker aldehydes, are actually intermediates of the Maillard reaction. The Maillard reaction, which involves mainly amino acids and reducing carbohydrates, but also other food ingredients such as lipids and polyphenols, leads to the formation of a great number of compounds with distinctive aromas characterized by a low odor threshold [33,34]. Among the identified volatiles in the five samples of bell pepper powder, phenylacetaldehyde (peak n°10) is the Strecker aldehyde deriving from phenylalanine [35], while 2-acetyl pyrrole (peak n°11) is a volatile Maillard reaction product deriving mainly from the reaction of glycine or valine with a reducing sugar [36]. Furthermore, 1-acetyl-pyrrolidine (peak n°18) has been reported as the end product of Maillard reaction involving proline [37]. Another Maillard reaction product is 3-hydroxy-2,3-dihydromaltol (peak n°17), which has been described as a caramelized-smelling compound [38]. Further thermal degradation products are 2-acetyl furan and 5-ethylfurfural (peak n°3 and 8), deriving from sugars [39] and 2-pentyl furan originated by lipid degradation [40].

In Table 2, the 16 identified terpene related compounds are highlighted by “b”apex. The terpenes accounts for a total area of ca 20% in the Senise samples, followed by paprika where they represent about 18% of the total chromatographic area. Their contribution to the aroma is much lower in the Altino samples with a maximum of 10% in Altino 2 and only 3% in Altino 3. The most abundant terpene in all the five spices is eremophila-9(10),11(12)-diene, which belongs to the family of eremophilane sesquiterpenes, together with eremophilene, the third terpene in order of abundance in the spices here investigated. It is important to underline that capsidiol, the principal phytoalexin of C. annuum, is an eremophilane-type sesquiterpenes as well [41]. *p*-Cymene (peak n°7), which is absent or present in traces in all the varieties excepting paprika where it represents ca 1.4% of the total area, can be formed by thermally induced isomerization and oxidation of monoterpene hydrocarbons [42]. The terpenes 3-carene, (−)-limonene, (+)-carvone, *β*-bisabolene, and *β*-sesquiphellandrene have been detected only in paprika. 

About ten of the 59 identified compounds listed in Table 2 are alkanes. Among the linear ones, tetradecane (peak n°33, unequivocally identified by GC/MS analysis of the authentic standard) is the most abundant in paprika, representing about 24% of the total area, while in both Altino and Senise bell pepper spices, it barely reaches 1%. Estragole (peak n°26) is present only in paprika with a relative abundance of about 1.4%. This is a phenylpropene derivative with an anise flavor found in anise, but also in many spices including chili paprika [43].

### 2.3. Classification 

Prior to the classification analysis, the available chromatograms have been divided into a training (66 samples) and a test set (30 samples) to enable the external validation of the models. Partitioning the objects in the two sets, special attention has been put in organizing the samples from Altino. In fact, in order to test the robustness of the model, all the Altino 2 and Altino 3 samples were included in the validation set. For what concerns all the other samples (pepper bell spices from Senise, Altino 1, and paprika), these were sub-divided by means of the Duplex algorithm [44].

Two different pre-processings of the training chromatograms, namely mean-centering and autoscaling, were tested. Thereafter, two different cross-validated PLS-DA models were built, and the optimal one was chosen looking at the cross-validated correct classification rates (%CR_cv_). As it can be appreciated by Table 3, the model created on auto-scaled data provided the lowest classification error, leading to a 100% CR_cv_. 

The application of this classification model to the test set provided excellent results for all classes; in fact, all the test samples were correctly classified. This is particularly relevant in the case of the Class Altino, because all the objects belonging to this category have been correctly assigned, regardless that samples were provided by different farmers. 

**Figure 2:**
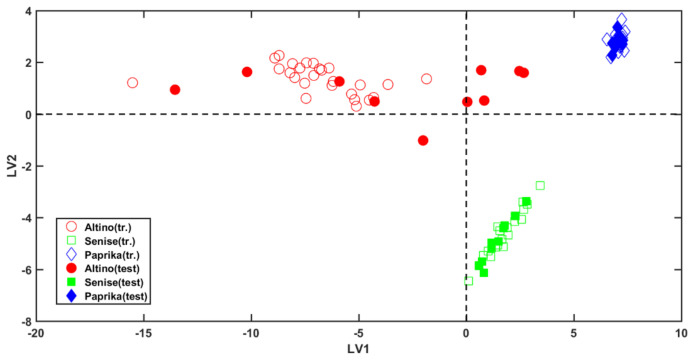
PLS-DA analysis: Score Plot. Empty and filled symbols represent training (tr.) and test samples, respectively.

A scores plot depicting the outcome of the PLS-DA analysis is shown in Figure 2. 

In the plot, empty and filled symbols represent training and test samples, respectively. Inspecting the figure, it is possible to appreciate that the samples show a clear grouping tendency according to the category they belong to. In particular, the first latent variable (LV1) allows the discrimination of samples belonging to class Paprika (blue diamonds) and to class Senise (green squares), which fall at positive values of this component, from those produced in Altino (red circles), presenting negative, or close to zero scores. On the other hand, bell peppers from Senise discern from all the others along the second latent variable (LV2); in fact, samples belonging to this category present negative scores, in opposition to all the other investigated objects, which fall at positive values of LV2.

In general, Class Paprika and Class Senise show a narrow within-class variance, whereas the Altino category has a wider one. As mentioned above, this is probably imputable to the fact the samples belonging to this class came from different producers, and probably they underwent slightly different procedures. 

With the intent of having a deeper insight into the model, Variable Importance in Projection (VIP) analysis [45] was used to understand which volatile compounds contribute the most to the solution of the classification problem, i.e., to the discrimination of the three different categories of bell pepper spices. As customarily advised, variables presenting a VIP index higher than 1 are considered as the most relevant. This approach led to the selection of the sixteen compounds reported in Table 4. 

**Figure 3:**
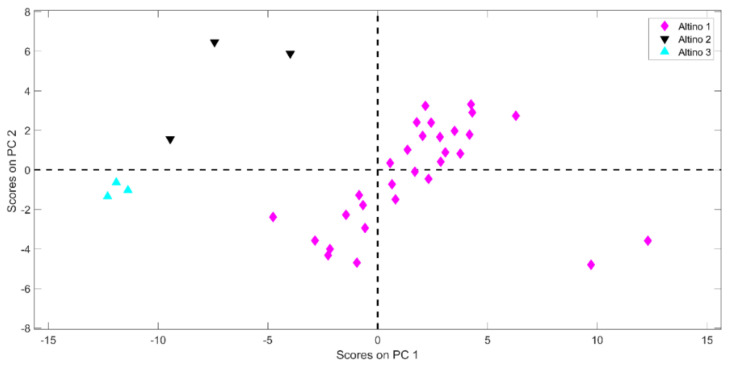
PC-plot. Projection of the Altino samples onto the space spanned by the first two PCs.

In order to determine whether there are any actual differences within the three lots of the Altino category, a Principal Component Analysis (PCA) was conducted only on the samples harvested in this area. This approach highlighted a relatively large variability within the class itself. Looking at the PC-plot displayed in Figure 3, it is apparent that the objects belonging to the three producers spread along PC1, showing a partial overlapping between samples belonging to class Altino 2 (black downward triangles) and Altino 3 (cyan triangles), which fall at negative scores of this component. Nevertheless, a partial division of these two categories is noticeable along PC2. In the PC1-PC2 plane, the samples are mainly grouped according to the manufacturer, despite the classes Altino 1 and Altino 2 exhibiting a relatively large internal variability. Given the narrowness of the area in which the Altino bell peppers are grown, the divergences among the samples produced by different farmers are not attributable to climatic conditions, but rather to slightly different productive processes (e.g., diversified drying settings). This observation highlights the goodness of the PLS-DA model built, which manages to efficiently discriminate the individuals belonging to this category despite the differences inherent in it. 

### 2.4. Discussion over the Comparison of the Outcome with the Literature 

The proposed approach proved to be suitable to trace the origin of bell pepper spices with recognized geographical identity. As described above, from the classification perspective, the results were definitely excellent. 

With the intention of comparing the outcome of this strategy with similar published works, it is straightforward that the juxtaposition falls on studies where these fruits have been classified by discriminant methods. In the literature, different works aim at the characterization/authentication of specific, high-valued, bell peppers. Many of them, probably due to a wider geographical spread of this product, focus on paprika. Among these, the closest to the present work is the one published by Arrizabalaga-Larrañaga and collaborators [11], who exploited the composition of capsaicinoids and carotenoids obtained by ultra-high-performance liquid chromatography-high-resolution mass spectrometry (UHPLC-HRMS) and PLS-DA, in order to discriminate paprika La Vera PDO, Murcia PDO, and common paprika from Czech Republic. The analysis was circumscribed to four capsaicinoids (nordihydrocapsaicin, capsaicin, dihydrocapsaicin, nordihydrocapsiate) and six carotenoids (capsanthin, capsorubin, violaxanthin, lutein, β‒cryptoxanthin, β‒carotene). The proposed UHPLC-HRMS-based strategy achieved the goal, reaching a classification rate of 80.9%. A similar work was conducted by Barbosa et al. [10], who coupled UHPLC-HRMS with PLS-DA in order to discriminate the same classes of paprika. The metabolomic fingerprint, coupled with PLS-DA, resulted in a suitable strategy, leading to a correct classification rate of 100%. 

Additionally, spectroscopy has also been used for the characterization of bell peppers. This is the case of the work published by Monago-Maraña and collaborators [7], who developed a chemometric-based spectroscopic tool for recognizing adulteration on a Spanish PDO paprika (*Pimentón de La Vera*). In their valuable work, the analytical technique used is Vis-NIR spectroscopy coupled with different classifiers (PLS-DA, PCA-linear discriminant analysis, and PCA-quadratic discriminant analysis). In agreement with the classification strategy we are proposing, the best results (in terms of correct classification rate on the external set of samples) were obtained when the classifier is PLS-DA (reaching 95% accuracy). A similar work was published by Biancolillo et al. [8], which aimed at the spectroscopic characterization of the Senise bell pepper, and at determining its possible adulteration with paprika. In this case, the ground samples were analyzed by Near and Mid Infrared spectroscopies. The best solution for the determination of the adulterant was carried out by means of a multi-block approach (SO-CovSel-LDA), which allowed obtaining 100% of correct classification on the external test set. 

In general, from the predictive point of view, even if the purpose of the classification models is slightly different, and therefore not completely comparable, the mentioned works have reported an equal (100%) or lower accuracy to the one obtained by the strategy we propose. With respect to this, the spectroscopic-based works present a clear advantage, i.e., they exploit a faster analytical method than SPME/GC-MS. Nevertheless, from the interpretative point of view, GC-MS allows collecting more detailed information on the composition of the inspected individuals. In fact, spectroscopic approaches lead to a qualitative establishment of the classes of compounds present in the samples, whereas, by means of GC-MS, we have been able to identify numerous volatile compounds present in the investigated bell peppers. 

Regarding the volatiles profile, it can be observed that the number of identified compounds is in agreement with similar works. The volatile composition of bell peppers has not been widely studied yet, consequently, there are not many comparisons to be made with the literature. In [20], the volatile fraction of fruits belonging to *capsicum annuum*, *chinense* and *frutescens* was investigated using SPME/GC-MS. A total of 13 volatile compounds, 7 sesqui- and mono-terpenes (*p*-cymene, (−)-limonene, *α*- and *β*-copaene, *β*-elemene, *α*-selinene, *β*-bisaboleneb), plus 6 other substances (Hexanal, 6-methyl-5-hepten-2-one, 2-pentyl-furan, *β*-cyclocitral, α- and *β*-ionone) are in common between the two works. More related details on this regard, together with a discussion on their agreement with the literature, is given in Section 2.2.

## 3. Materials and Methods

### 3.1. Materials and Samples

The bell pepper spices analyzed in this work, all in the dried and ground form, were produced in 2020. The samples were provided by the following manufacturers: A.C.A. Agrolearia Colline Altinensi, Altino (CH, Italy) (Altino 1); Azienda Agricola Terra Fonte, Altino (Altino 2); Azienda Agricola La Tavola dei Briganti, Altino (Altino 3); Masseria Agricola Buongiorno, Senise (PZ, Italy) (Senise); Fertitecnica Colfiorito, Colfiorito (PG, Italy) (paprika). Retention Index Standard (aliphatic C_7_-C_40_ hydrocarbons dissolved in hexane) was purchased from Sigma-Aldrich (Saint Louis, MO, USA). The numerosity of the analyzed objects (per manufacturer) has been reported above in Table 2. 

### 3.2. Headspace Solid-Phase Micro-Extraction (HS-SPME)

The SPME fiber used in this work was coated with divinylbenzene/carboxen/polydimethylsiloxane (DVB/CAR/PDMS), 50/30 μm thickness (Supelco, Bellefonte, PA, USA). The fiber was introduced in the vial equipped with the Mininert cap containing 200 mg of sample and exposed for 10 min to the head space of the sample kept in an oil bath at 90 °C under magnetic stirring. After extraction of the ground bell pepper volatiles, the fiber was inserted into the injection port of the GC apparatus where desorption took place at 250 °C for 2 min. After each analysis, the fiber was kept in the GC injection port at 270 °C for 5 min and after that a blank run was recorded to check the cleaning of the sorbent. 

### 3.3. Gas Chromatography-Mass Spectrometry (GC-MS) Analysis

All the analyses were carried out on a Saturn 2000 GC-MS instrument (Varian, Inc., Palo Alto, CA, USA). The GC apparatus was equipped with a 1078 split/splitless injector with a SPME liner inside. All injections were performed in split mode with a 10:1 split ratio.

A Varian FactorFour™ VF5-ms capillary column (5% phenyl-95% polymethylsiloxane, 30 m × 0.25 mm × 0.25 μm film thickness) was used and the carrier gas was helium IP supplied at a flow rate of 1.1 mL/min. GC separation was achieved using the following column oven temperature program: initial temperature 50 °C for 2 min, then increased to 90 °C at 25 °C/min, then to 128 °C at a rate of 6 °C/min and to 170 °C at 4 °C/min, at the end the temperature of 280 °C was reached at 75 °C/min and hold for 3.1 min. The EI-ion trap mass spectrometer operated at the following conditions: ionization energy 70 eV; 40–600 *m/z* range; 1 s scan cycle time; transfer line temperature 170 °C; manifold temperature 110 °C; ion trap temperature 150 °C. The compounds were identified by comparison of their linear retention indices calculated using a linear alkane mixture (C_7_-C_40_) and their mass spectra with those reported in standard libraries [46] or in the specialized literature [47].

### 3.4. DoE-Based Optimization of the HS-SPME/GC-MS Conditions

While running HS-SPME/GC-MS experiments, two factors were recognized as those influencing most the analysis: the exposure time of the fiber (*t*) and the temperature (*T*) of the sample. Since each variable has been investigated at two different levels, a design of the experiment (DoE) 2^*k*^ was exploited. Including three replicated measures of the central point of the DoE, seven (=2^2^ + 3) experiments were designed. 

The model assumed to be suitable for describing the investigated system is expressed by Equation (2):

(2) $$Y=b_{0}+b_{1}x_{1}+b_{2}x_{2}+b_{12}x_{1}x_{2}$$

where $x_{1}\;$= t and $x_{2}\;$= T.

The response *Y* corresponds to the logarithm of the total area (*A*) of the chromatograms (RT between 5 and 19 min) after the 10 peaks with the largest area having been eliminated. Given these premises, the optimal chromatographic conditions are those allowing the maximization of log*A*. All the calculation related to the DoE were run by means of the CAT (chemometric Agile Tool) software [48].

### 3.5. Chemometric Methods 

In the present work, Partial Least Squares Discriminant Analysis (PLS-DA) [23,49] was used as discriminant classifier. This approach has been developed as a direct extension of the Linear Discriminant Analysis (LDA) [50] and it was conceived to overcome the issues associated to the non-invertibility of the variance–covariance matrix. 

PLS-DA is based on the possibility of transforming a classification problem into a regression one thanks to the mediation of a *dummy Y* response codifying the class-membership [51]. Basically, each individual can be associated to a binary y-vector encoding the class-information. For instance, for a three-category case, samples belonging to class A, class B, and class C will be identified by the vectors $y_{A}=\left[1\;0\;0\right]$, $y_{B}=\left[0\;1\;0\right]$, and $y_{C}=\left[0\;0\;1\right]$, respectively. This allows the creation (and the subsequent solution) of a classification problem solvable by means of PLS. Once the calibration model is built and the regression coefficients estimated, new samples can be classified. The application of the model on a novel set of observations provides a continuous, non-categorical, $\hat{Y}$. The association of novel samples to the different classes can be carried out in different ways [52,53,54].

One of the benefits of PLS-DA is that, due to its strict link with PLS, Variable Importance in Projection (VIP) [45] analysis can be used to estimate which analytes characterize the different classes. In fact, this method, conceived as a feature selection approach to be used in combination with PLS modelling, allows evaluating which feature greatly contributes to the regression. In particular, a VIP index is calculated for each modelled variable; customarily, features presenting a VIP index >1 are considered the most relevant [55]. PLS-DA was performed using in-house routines in the MATLAB environment (R2019b; The Mathworks, Natick, MA, USA).

## 4. Conclusions

The aroma profile of bell pepper spice, collected by HS-SPME/GC-MS and classified by PLS-DA, offers a powerful tool for the identification of the origin of this spice.

The optimized HS-SPME/GC-MS procedure allowed the identification of 59 volatile compounds. VIP analysis, subsequent to the PLS-DA model, has revealed which compounds contribute the most to the discrimination of the three classes.

In general, the proposed strategy demonstrated to definitely be a suitable solution for tracing bell peppers and discriminating them according to their geographical origin; in fact, it allowed the correct classification of all the validation samples, reaching a 100% of accuracy in prediction.

In conclusion, the present study has achieved different milestones.

From the analytical point of view, it made it possible to obtain the volatile profile of three different classes of bell peppers, on which, despite the recognized geographical identity and the quality labels (P.G.I and Slow Food presidia), this process had not been completed yet.

Furthermore, it provides some benefits from the legal and the economic standpoint. In fact, it has shown that the coupling of HS-SPME/GC-MS and PLS-DA can be used to trace bell pepper spices. This aspect has several implications, since it lays the foundations for the development of a method that allows defining and checking the geographical origin of this kind of product, representing a valid tool for its quality control. It is straightforward that, due to the market-value of aliments presenting a quality mark, this achievement is of particular interest both from the juridical and commercial perspectives, ensuring consumers’ protection from possible frauds.

## Figures and Tables

**Table 1:** Synopsis of the applied Design of the Experiment, variables (temperature and time), levels, and response (log*A*).

*T* (°C)-*t* (min)	*T* Level	*t* Level	log*A*
90-10	+1	−1	5.669
70-10	−1	−1	5.564
90-30	+1	+1	5.733
70-30	−1	+1	6.029
80-20	0	0	5.732 ± 0.035 ^a^

^a^ mean value ± standard deviation (*n* = 3).

**Table 2:** Volatile profiles of bell pepper spices: peak number; observed retention time (RT (min)), assigned chemical structure and mean relative (%) peak area in the GC/MS chromatograms with related standard deviation for each variety: Altino bell pepper (from three different manufactures: 1, 2, and 3), Senise bell pepper, and commercial paprika.

Peak n°	RT (min)	Compound	Bell Pepper Spices				
			Altino 1 (*n* = 30)	Altino 2 (*n* = 3)	Altino 3 (*n* = 3)	Senise (*n* = 30)	Paprika (*n* = 30)
1	3.41	hexanal	0.20 ± 0.05	0.35 ± 0.33	0.84 ± 0.85	0.23 ± 0.13	0.07 ± 0.10
2	4.11	fatty acid	0.14 ± 0.03	n.d.	0.31 ± 0.03	0.12 ± 0.03	n.d.
3	4.60	2-acetylfuran	1.04 ± 0.24	0.26 ± 0.08	0.85 ± 0.09	1.65 ± 0.34	0.47 ± 0.07
4	5.54	6-methyl-5-hepten-2-one ^a^	0.58 ± 0.20	0.43 ± 0.28	0.38 ± 0.07	0.57 ± 0.12	0.58 ± 0.11
5	5.63	2-pentyl-furan	0.91 ± 0.25	1.12 ± 0.85	0.70 ± 0.14	1.01 ± 0.24	0.64 ± 0.09
6	5.97	3-carene ^b^	n.d.	n.d.	n.d.	n.d.	2.79 ± 0.52
7	6.20	*p*-cymene ^b^	0.01 ± 0.01	n.d.	n.d.	0.04 ± 0.03	1.42 ± 0.26
8	6.26	5-ethylfurfural	0.35 ± 0.10	0.62 ± 0.18	0.15 ± 0.03	0.69 ± 0.15	0.65 ± 0.13
9	6.27	(−)-limonene ^b^	n.d.	n.d.	n.d.	n.d.	2.26 ± 0.81
10	6.54	phenylacetaldehyde	1.57 ± 0.35	5.46 ± 1.44	10.12 ± 0.48	4.63 ± 0.54	3.86 ± 0.45
11	6.77	2-acetylpyrrole	0.51 ± 0.09	0.27 ± 0.10	0.08 ± 0.14	0.22 ± 0.05	0.45 ± 0.08
12	6.87	(E.E)-3.5-octadien-2-one	2.40 ± 0.63	0.32 ± 0.24	0.53 ± 0.10	0.92 ± 0.17	0.55 ± 0.10
13	6.96	4-methyl-benzaldehyde	0.77 ± 0.18	1.60 ± 0.51	0.54 ± 0.05	1.68 ± 0.24	0.97 ± 0.10
14	7.28	(E.E)-3.5-nonadien-2-one	1.35 ± 0.34	0.11 ± 0.04	0.36 ± 0.18	0.35 ± 0.08	0.11 ± 0.02
15	7.45	6-methyl-3.5-heptadien-2-one ^a^	6.13 ± 1.47	7.52 ± 1.60	5.33 ± 0.67	11.54 ± 1.82	8.21 ± 0.76
16	7.66	2.6-dimethyl-cyclohexanol	2.64 ± 0.64	2.95 ± 1.03	3.10 ± 0.32	2.50 ± 0.50	2.89 ± 0.37
17	8.20	3-hydroxy-2.3-dihydromaltol	2.41 ± 0.52	10.51 ± 4.08	8.33 ± 0.63	4.72 ± 1.33	6.39 ± 1.09
18	8.59	1-acetyl-pyrrolidine	2.53 ± 0.24	0.30 ± 0.18	0.19 ± 0.02	0.59 ± 0.09	0.29 ± 0.14
19	8.88	2.4-dimethyl-benzaldehyde	2.30 ± 0.37	4.29 ± 0.59	1.65 ± 0.18	4.93 ± 0.39	2.52 ± 0.12
20	9.35	safranal ^a^	1.26 ± 0.31	5.32 ± 1.21	2.54 ± 0.24	3.11 ± 0.45	4.51 ± 0.48
21	9.63	*m*-cumenol	0.17 ± 0.11	0.27 ± 0.04	0.18 ± 0.13	0.47 ± 0.05	0.19 ± 0.04
22	9.74	*β*-cyclocitral ^a^	1.38 ± 0.31	1.23 ± 0.26	0.88 ± 0.07	2.25 ± 0.32	1.15 ± 0.10
23	10.21	(+)-carvone ^b^	n.d.	n.d.	n.d.	n.d.	1.00 ± 0.05
24	10.54	trans-9-decalol	0.20 ± 0.05	0.05 ± 0.01	0.14 ± 0.00	0.03 ± 0.02	n.d.
25	10.63	branched alkane	0.08 ± 0.11	0.02 ± 0.02	0.13 ± 0.03	0.08 ± 0.03	0.13 ± 0.02
26	11.09	estragole	n.d.	n.d.	n.d.	n.d.	1.37 ± 0.06
27	11.56	2-(2-methylpropylidene)-cycloheptanone	0.14 ± 0.01	0.15 ± 0.02	0.30 ± 0.01	0.12 ± 0.01	0.13 ± 0.01
28	11.67	branched alkane	0.36 ± 0.10	0.03 ± 0.01	0.17 ± 0.03	0.06 ± 0.01	0.16 ± 0.02
29	11.94	fumaric acid. di ester	2.75 ± 0.48	0.19 ± 0.07	2.94 ± 0.45	0.38 ± 0.10	n.d.
30	12.66	branched alkane	2.28 ± 0.29	3.38 ± 0.54	0.94 ± 0.07	1.15 ± 0.10	n.d.
31	13.15	*α*-copaene ^b^	0.41 ± 0.06	0.27 ± 0.06	0.33 ± 0.09	2.01 ± 0.17	0.59 ± 0.04
32	13.43	*β*-elemene	1.11 ± 0.08	1.87 ± 0.32	0.21 ± 0.04	3.44 ± 0.22	1.19 ± 0.06
33	13.54	tetradecane	0.34 ± 0.03	0.98 ± 0.23	1.14 ± 0.11	1.19 ± 0.11	23.92 ± 0.90
34	14.19	*α*-ionone ^a^	1.11 ± 0.04	0.58 ± 0.08	1.55 ± 0.04	0.87 ± 0.06	n.d.
35	14.23	*β*-copaene ^b^	0.66 ± 0.04	0.39 ± 0.03	0.89 ± 0.03	0.58 ± 0.11	1.14 ± 0.06
36	14.48	dihydro-*β*-ionone ^a^	0.36 ± 0.02	0.50 ± 0.07	0.57 ± 0.06	0.39 ± 0.05	0.36 ± 0.05
37	14.63	alkene/alcohol	0.79 ± 0.04	1.42 ± 0.24	3.29 ± 0.14	0.43 ± 0.07	n.d.
38	14.76	dihydropseudoionone ^a^	11.68 ± 0.68	3.61 ± 0.49	7.23 ± 0.18	7.11 ± 0.72	6.42 ± 0.54
39	14.90	aristolene ^b^	0.22 ± 0.22	0.45 ± 0.06	0.67 ± 0.02	0.81 ± 0.17	0.56 ± 0.06
40	15.10	branched alkane	2.90 ± 0.13	5.65 ± 0.96	1.43 ± 0.04	1.44 ± 0.06	0.14 ± 0.03
41	15.26	dehydro-isolongifolene ^b^	0.64 ± 0.03	0.83 ± 0.15	n.d.	1.99 ± 0.09	n.d.
42	15.53	*γ*-selinene ^b^	0.49 ± 0.02	0.81 ± 0.12	n.d.	1.56 ± 0.07	1.00 ± 0.06
43	15.62	*β*-ionone ^a^	19.12 ± 1.31	7.39 ± 1.06	11.86 ± 0.59	10.69 ± 1.20	5.88 ± 0.65
44	15.72	*β*-ionone epoxide ^a^	7.77 ± 0.88	2.41 ± 0.30	13.54 ± 0.51	4.01 ± 0.49	2.03 ± 0.24
45	15.81	eremophila-9(10).11(12)-diene ^b^	1.80 ± 0.07	2.58 ± 0.47	0.35 ± 0.03	5.16 ± 0.20	2.08 ± 0.10
46	15.99	eremophilene ^b^	0.49 ± 0.05	1.06 ± 0.18	0.10 ± 0.01	1.74 ± 0.11	1.47 ± 0.10
47	16.14	*α*-selinene ^b^	0.41 ± 0.02	1.00 ± 0.12	0.10 ± 0.00	1.58 ± 0.07	1.66 ± 0.10
48	16.32	*β*-bisabolene ^b^	n.d.	n.d.	n.d.	n.d.	0.38 ± 0.05
49	16.43	guaia-1(10).11-diene ^b^	0.42 ± 0.06	0.52 ± 0.11	0.05 ± 0.02	1.46 ± 0.10	0.10 ± 0.02
50	16.66	methyl dodecanoate	0.38 ± 0.04	0.48 ± 0.08	0.59 ± 0.06	0.73 ± 0.09	0.51 ± 0.05
51	16.75	*β*-sesquiphellandrene ^b^	n.d.	n.d.	n.d.	n.d.	0.34 ± 0.06
52	16.84	linear alkane	0.21 ± 0.06	0.08 ± 0.03	0.22 ± 0.02	0.08 ± 0.02	n.d.
53	17.02	dihydroactinidiolide ^a^	9.37 ± 1.64	5.40 ± 1.56	8.83 ± 0.80	6.10 ± 1.17	4.39 ± 0.60
54	17.66	branched alkane	0.82 ± 0.07	1.75 ± 0.29	0.59 ± 0.04	0.40 ± 0.04	0.11 ± 0.03
55	18.62	branched alkane	0.97 ± 0.12	3.05 ± 0.71	1.79 ± 0.09	0.57 ± 0.08	0.45 ± 0.08
56	19.39	occidentalol ester	0.64 ± 0.13	n.d.	0.04 ± 0.01	0.21 ± 0.08	n.d.
57	20.24	branched alkane	0.97 ± 0.14	3.48 ± 0.85	0.86 ± 0.06	0.36 ± 0.08	0.10 ± 0.04
58	20.84	heptadecane	1.14 ± 0.20	6.08 ± 0.78	1.39 ± 0.09	0.42 ± 0.07	0.36 ± 0.10
59	21.09	methyl tetradecanoate	0.33 ± 0.06	0.61 ± 0.17	0.68 ± 0.09	0.65 ± 0.14	0.81 ± 0.13

^a^ derived from degradation of carotenoids; ^b^ terpene.

**Table 3:** PLS-DA analysis. Pre-processing, number of latent variables (LVs), and average cross-validated classification rates.

Pre-Processing	LVs	% CR_cv_
Mean-centering	1	91.1
Auto-scaling	2	100.0

**Table 4:** VIP analysis: compounds presenting a VIP index >1.

Compound
3-carene
*p*-cymene
(−)-limonene
(+)-carvone
estragole
*α*-copaene
*β*-elemene
tetradecane
*β*-copaene
dehydro isolongifolene
*γ*-selinene
eremophila-9(10),11(12)-diene
eremophilene
*β*-bisabolene
guaia-1(10),11-diene
*β*-sesquiphellandrene

## Sample Availability

Samples of the compounds are not available from the authors.

## References

[1] de Sá Mendes N., de Andrade Gonçalves É.C.B. (2020). The role of bioactive components found in peppers. Trends Food Sci. Technol..

[2] Daood H.G., Palotás G., Palotás G., Somogyi G., Pék Z., Helyes L. (2014). Carotenoid and antioxidant content of ground paprika from indoor-cultivated traditional varieties and new hybrids of spice red peppers. Food Res. Int..

[3] Speranza G., Lo Scalzo R., Morelli C.F., Rabuffetti M., Bianchi G. (2019). Influence of drying techniques and growing location on the chemical composition of sweet pepper (*Capsicum annuum* L., var. Senise). J. Food Biochem..

[4] Martín A., Hernández A., Aranda E., Casquete R., Velázquez R., Bartolomé T., Córdoba M.G. (2017). Impact of volatile composition on the sensorial attributes of dried paprikas. Food Res. Int..

[5] Galvin-King P., Haughey S.A., Elliott C.T. (2018). Herb and spice fraud; the drivers, challenges and detection. Food Control.

[6] Ballin N.Z., Laursen K.H. (2019). To target or not to target? Definitions and nomenclature for targeted versus non-targeted analytical food authentication. Trends Food Sci. Technol..

[7] Monago-Maraña O., Eskildsen C.E., Galeano-Díaz T., de la Peña A.M., Wold J.P. (2021). Untargeted classification for paprika powder authentication using visible—Near infrared spectroscopy (VIS-NIRS). Food Control.

[8] Biancolillo A., Di Donato F., Merola F., Marini F., D’Archivio A.A. (2021). Sequential data fusion techniques for the authentication of the P.G.I. senise (“crusco”) bell pepper. Appl. Sci..

[9] Campmajó G., Rodríguez-Javier L.R., Saurina J., Núñez O. (2021). Assessment of paprika geographical origin fraud by high-performance liquid chromatography with fluorescence detection (HPLC-FLD) fingerprinting. Food Chem..

[10] Barbosa S., Saurina J., Puignou L., Núñez O. (2020). Classification and authentication of paprika by UHPLC-HRMS fingerprinting and multivariate calibration methods (PCA and PLS-DA). Foods.

[11] Arrizabalaga-Larrañaga A., Campmajó G., Saurina J., Núñez O., Santos F.J., Moyano E. (2021). Determination of capsaicinoids and carotenoids for the characterization and geographical origin authentication of paprika by UHPLC–APCI–HRMS. LWT.

[12] Mudrić S., Gašić U.M., Dramićanin A.M., Ćirić I., Milojković-Opsenica D.M., Popović-Đorđević J.B., Momirović N.M., Tešić Ž.L. (2017). The polyphenolics and carbohydrates as indicators of botanical and geographical origin of Serbian autochthonous clones of red spice paprika. Food Chem..

[13] Fiamegos Y., Dumitrascu C., Papoci S., de la Calle M.B. (2021). Authentication of PDO paprika powder (Pimentón de la Vera) by multivariate analysis of the elemental fingerprint determined by ED-XRF. A feasibility study. Food Control.

[14] Portis E., Nervo G., Cavallanti F., Barchi L., Lanteri S. (2006). Multivariate analysis of genetic relationships between Italian pepper landraces. Crop Sci..

[15] Manzi A. (2006). Origine e Storia Delle Piante Coltivate in Abruzzo.

[16] D’Archivio A.A., Di Pietro L., Maggi M.A., Rossi L. (2018). Optimization using chemometrics of HS-SPME/GC–MS profiling of saffron aroma and identification of geographical volatile markers. Eur. Food Res. Technol..

[17] Xu C.H., Chen G.S., Xiong Z.H., Fan Y.X., Wang X.C., Liu Y. (2016). Applications of solid-phase microextraction in food analysis. TrAC Trends Anal. Chem..

[18] Souza-Silva É.A., Gionfriddo E., Pawliszyn J. (2015). A critical review of the state of the art of solid-phase microextraction of complex matrices II. Food analysis. TrAC Trends Anal. Chem..

[19] Cremer D.R., Eichner K. (2000). Formation of volatile compounds during heating of spice paprika (*Capsicum annuum*) powder. J. Agric. Food Chem..

[20] Rodríguez-Burruezo A., Kollmannsberger H., González-Mas M.C., Nitz S., Fernando N. (2010). HS-SPME comparative analysis of genotypic diversity in the volatile fraction and aroma-contributing compounds of capsicum fruits from the annuum? chinense? Frutescens complex. J. Agric. Food Chem..

[21] Rios J.J., Fernández-García E., Mínguez-Mosquera M.I., Pérez-Gálvez A. (2008). Description of volatile compounds generated by the degradation of carotenoids in paprika, tomato and marigold oleoresins. Food Chem..

[22] Cirlini M., Luzzini G., Morini E., Folloni S., Ranieri R., Dall’Asta C., Galaverna G. (2019). Evaluation of the volatile fraction, pungency and extractable color of different Italian Capsicum annuum cultivars designed for food industry. Eur. Food Res. Technol..

[23] Sjöström M., Wold S., Söderström B. (1986). PLS discriminant plots. Pattern Recognition in Practice.

[24] Di Donato F., Biancolillo A., Mazzulli D., Rossi L., D’Archivio A.A. (2021). HS-SPME/GC–MS volatile fraction determination and chemometrics for the discrimination of typical Italian Pecorino cheeses. Microchem. J..

[25] De Luca S., Ciotoli E., Biancolillo A., Bucci R., Magrì A.D., Marini F. (2018). Simultaneous quantification of caffeine and chlorogenic acid in coffee green beans and varietal classification of the samples by HPLC-DAD coupled with chemometrics. Environ. Sci. Pollut. Res..

[26] Giannetti V., Boccacci Mariani M., Marini F., Biancolillo A. (2021). Effects of thermal treatments on durum wheat pasta flavour during production process: A modelling approach to provide added-value to pasta dried at low temperatures. Talanta.

[27] Liang J., Sun J., Chen P., Frazier J., Benefield V., Zhang M. (2021). Chemical analysis and classification of black pepper (*Piper nigrum* L.) based on their country of origin using mass spectrometric methods and chemometrics. Food Res. Int..

[28] Torres-Cobos B., Quintanilla-Casas B., Romero A., Ninot A., Alonso-Salces R.M., Toschi T.G., Bendini A., Guardiola F., Tres A., Vichi S. (2021). Varietal authentication of virgin olive oil: Proving the efficiency of sesquiterpene fingerprinting for Mediterranean Arbequina oils. Food Control.

[29] Teribia N., Buvé C., Bonerz D., Aschoff J., Hendrickx M., Loey A.V. (2021). Effect of cultivar, pasteurization and storage on the volatile and taste compounds of strawberry puree. LWT.

[30] Di Donato F., D’Archivio A.A., Maggi M.A., Rossi L. (2021). Detection of Plant-Derived Adulterants in Saffron (*Crocus sativus* L.) by HS-SPME/GC-MS Profiling of Volatiles and Chemometrics. Food Anal. Methods.

[31] Winterhalter P., Rouseff R. (2001). Carotenoid-Derived Aroma Compounds: An Introduction.

[32] Krammer G.E., Werkhoff P., Sommer H., Schmidt C.O., Gatfield I., Bertram H.J. (2001). Carotenoid Degradation Products in Paprika Powder.

[33] Mlotkiewicz J.A., O’Brien J., Nursten H.E., Crabbe M.J.C., Ames J.M. (1988). The role of the Maillard reaction in the food industry. The Maillard Reaction in Foods and Medicine.

[34] Nagodawithana T.W., Nagodawithana T.W. (1995). Maillard and other flavor producing reactions. Savory Flavors.

[35] Buttery R.G., Ling L.C. (1993). Volatile Components of Tomato Fruit and Plant Parts, Relationship and Biogenesis. Bioactive Volatile Compounds from Plants.

[36] Cho I.H., Lee S., Jun H.R., Roh H.J., Kim Y.S. (2010). Comparison of volatile Maillard reaction products from tagatose and other reducing sugars with amino acids. Food Sci. Biotechnol..

[37] Yaylayan V.A., Keyhani A., Huygues-Despointes A., Shahidi F., Ho C.T., van Chuyen N. (1998). Generation and the fate of C2, C3 and C4 reactive fragments formed in Maillard model systems of [13C] glucose and [13C]glycine or proline. Process-Induced Chemical Changes in Food. Advances in Experimental Medicine and Biology.

[38] Preininger M., Gimelfarb L., Li H.C., Dias B.E., Fahmy F., White J. (2009). Identification of dihydromaltol (2,3-dihydro-5-hydroxy6-methyl-4h-pyran-4- one) in ryazhenka kefir and comparative sensory impact assessment of related cycloenolones. J. Agric. Food Chem..

[39] Chuyen N.V., Shahidi F., Ho C.T., van Chuyen N. (1998). Maillard Reaction and Food Processing. Process-Induced Chemical Changes in Food. Advances in Experimental Medicine and Biology.

[40] Jennings W.G., Shibamoto T. (1980). Qualitative Analysis of Flavour and Fragrance Volatiles by Glass Capillary Gas Chromatographye.

[41] Zhao Y., Schenk D.J., Takahashi S., Chappell J., Coates R.M. (2004). Eremophilane sesquiterpenes from capsidiol. J. Org. Chem..

[42] Wrolstad R.E., Jennings W.G. (1965). Chromatostripisomerization of terpenes. J. Chromatogr. A.

[43] Silvis I.C.J., Luning P.A., Klose N., Jansen M., van Ruth S.M. (2019). Similarities and differences of the volatile profiles of six spices explored by Proton Transfer Reaction Mass Spectrometry. Food Chem..

[44] Snee R.D. (1977). Validation of Regression Models: Methods and Examples. Technometrics.

[45] Wold S., Johansson E., Cocchi M. (1993). PLS—Partial least-squares projections to latent structures. 3D QSAR Drug Design.

[46] (2014). NIST14: Mass Spectral Database.

[47] Adams R.P. (2007). Identification of Essential Oil Components by Gas Chromatography/Mass Spectrometry.

[48] Leardi R., Melzi C., Polotti G. CAT (Chemometric Agile Tool). http://www.gruppochemiometria.it.

[49] Biancolillo A., Marini F., Ruckebusch C., Vitale R. (2020). Chemometric strategies for spectroscopy-based food authentication. Appl. Sci..

[50] Fischer R.A. (1936). The use of multiple measurements in taxonomic problems. Ann. Eugen..

[51] Barker M., Rayens W. (2003). Partial least squares for discrimination. J. Chemom..

[52] Indahl U.G., Martens H., Næs T. (2007). From dummy regression to prior probabilities in PLS-DA. J. Chemom..

[53] Nocairi H., Qannari E.M., Vigneau E., Bertrand D. (2005). Discrimination on latent components with respect to patterns. Application to multicollinear data. Comput. Stat. Data Anal..

[54] Pérez N.F., Ferré J., Boqué R. (2009). Calculation of the reliability of classification in discriminant partial least-squares binary classification. Chemom. Intell. Lab. Syst..

[55] Cocchi M., Biancolillo A., Marini F. (2018). Chemometric Methods for Classification and Feature Selection. Comprehensive Analytical Chemistry.

